# Mutations in the mitochondrial tryptophanyl‐tRNA synthetase cause growth retardation and progressive leukoencephalopathy

**DOI:** 10.1002/mgg3.654

**Published:** 2019-03-28

**Authors:** Camilla Maffezzini, Isabelle Laine, Cristina Dallabona, Paula Clemente, Javier Calvo‐Garrido, Rolf Wibom, Karin Naess, Michela Barbaro, Anna Falk, Claudia Donnini, Christoph Freyer, Anna Wredenberg, Anna Wedell

**Affiliations:** ^1^ Max Planck Institute Biology of Ageing ‐ Karolinska Institutet Laboratory Stockholm Sweden; ^2^ Department of Medical Biochemistry and Biophysics Karolinska Institutet Stockholm Sweden; ^3^ Department of Chemistry, Life Sciences and Environmental Sustainability University of Parma Parma Italy; ^4^ Department of Molecular Medicine and Surgery Karolinska Institutet Stockholm Sweden; ^5^ Centre for Inherited Metabolic Diseases Karolinska University Hospital Stockholm Sweden; ^6^ Department of Neuroscience Karolinska Institutet Stockholm Sweden

**Keywords:** aminoacylation, mitochondria, mitochondrial tryptophanyl‐tRNA synthetase, WARS2

## Abstract

**Background:**

Mutations in mitochondrial aminoacyl tRNA synthetases form a subgroup of mitochondrial disorders often only perturbing brain function by affecting mitochondrial translation. Here we report two siblings with mitochondrial disease, due to compound heterozygous mutations in the mitochondrial tryptophanyl‐tRNA synthetase (*WARS2*) gene, presenting with severe neurological symptoms but normal mitochondrial function in skeletal muscle biopsies and cultured skin fibroblasts.

**Methods:**

Whole exome sequencing on genomic DNA samples from both subjects and their parents identified two compound heterozygous variants c.833T>G (p.Val278Gly) and c.938A>T (p.Lys313Met) in the *WARS2* gene as potential disease‐causing variants. We generated patient‐derived neuroepithelial stem cells and modeled the disease in yeast and *Drosophila melanogaster* to confirm pathogenicity.

**Results:**

Biochemical analysis of patient‐derived neuroepithelial stem cells revealed a mild combined complex I and IV defect, while modeling the disease in yeast demonstrated that the reported aminoacylation defect severely affects respiration and viability. Furthermore, silencing of wild type *WARS2 *in *Drosophila melanogaster *showed that a partial defect in aminoacylation is enough to cause lethality.

**Conclusions:**

Our results establish the identified *WARS2* variants as disease‐causing and highlight the benefit of including human neuronal models, when investigating mutations specifically affecting the nervous system.

## 1 INTRODUCTION

The primary function of mitochondrial aminoacyl tRNA synthetases (ARS2) is to attach the correct amino acid to its corresponding tRNA through esterification; hydrolysing ATP to AMP in the process (Ibba & Soll, 2000). In mitochondria, 19 different ARS2 proteins aminoacylate the 22 mitochondrial DNA (mtDNA) encoded tRNAs. This limited redundancy means that defects in these genes will severely affect mitochondrial translation, oxidative phosphorylation, and ultimately cellular function. It is thus not surprising that numerous *ARS2* genes have been associated with mitochondrial diseases, presenting with a variety of clinical symptoms (Konovalova & Tyynismaa, 2013; Sissler, González‐Serrano, & Westhof, 2017). For instance, mutations in the mitochondrial aspartyl‐tRNA synthetase, DARS2 (MIM 610956), can lead to leukoencephalopathy (Scheper et al., 2007; van Berge et al., 2013), while dysfunctional leucyl‐tRNA synthetase, LARS2 (MIM 604544), has been linked to several pathogenic conditions including infertility and hearing loss (Pierce et al., 2013; Riley et al., 2016). Mutations in the mitochondrial tryptophanyl‐tRNA synthetase, WARS2 (MIM 604733), have been linked to developmental delay, intellectual disability, microcephaly, seizures, and brain atrophy (Burke et al., 2018; Musante et al., 2017; Theisen et al., 2017; Vantroys et al., 2018; Wortmann et al., 2017), but also with aggressive behavior (Musante et al., 2017) hepatopathy (Vantroys et al.,^2018^), or early onset Parkinsonism (Burke et al., 2018). In total, all *ARS2* genes have been associated with mitochondrial disease, with more than 150 different mutations reported to date (Sissler et al., 2017). And although, as true for many mitochondrial disorders, the central nervous system is predominantly affected, dysfunctions in other organs have also been reported and the clinical presentations can therefore be quite varying (Konovalova & Tyynismaa, 2013; Sissler et al., 2017).

We here identify two siblings carrying the previously reported c.928A>T (p.Lys313Met) (Theisen et al., 2017; Vantroys et al., 2018; Wortmann et al., 2017), and the novel c.833T>G (p.Val278Gly) variants as compound heterozygous mutations in the mitochondrial *WARS2* gene, presenting with growth retardation and progressive leukoencephalopathy/neurodegeneration with reduced supratentorial white matter volume. Biochemical investigations in patient muscle biopsies did not reveal any defect of the respiratory chain. To validate these variants, we expressed mutant alleles in the yeast system, reprogrammed patient fibroblasts to induced pluripotent stem (iPS) cells followed by differentiation to neuroepithelial‐like stem (NES) cells, and studied the consequences of reduced WARS2 expression in the fruit fly, *Drosophila melanogaster*. Together our results validate the identified variants as disease‐causing and highlight the importance of employing human neuronal models when investigating the pathogenicity of mutations specifically affecting the nervous system.

### 1.1 Subjects

**Figure 1:**
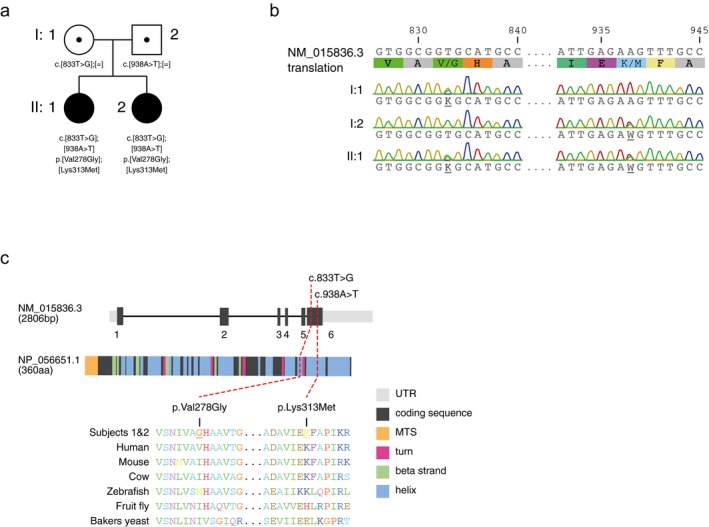
(a) Pedigree of the family affected by compound heterozygous mutations in *WARS2* (NM_015836.3). Mutation status of affected (closed symbols) and healthy carriers (open symbols with dot) are shown, together with the variants and affected amino acid position. (b) Electropherogram of both parents (I.1, I.2) as well as one affected individual (II.1). (c) Primary structure and cross species protein conservation of WARS2 (NP_056651.1).

Subject 1 (II:1) is the first daughter of healthy, nonconsanguineous parents of Swedish descent (Figure 1a). She was delivered by cesarean section at gestational week 37 due to intrauterine growth retardation (IUGR). Birth weight (BW) was 2,165 g; birth length (BL): 45 cm; and head circumference: 30 cm.

Psychomotor development was delayed from the first year of life. At 18 months of age she had general muscle weakness, hypotonia, and ataxia. Her tendon reflexes were brisk, indicating a central hypotonia. She later developed spasticity in arms and legs, with remaining pronounced axial hypotonia. Cardiac evaluation at the age of three years revealed a congenital atrial septum defect (ASD), which was later surgically corrected. At the age of eight years, epileptic seizures appeared. Cognitive development was impaired. At the current age of 15 years, the girl has a moderate–severe intellectual disability and attends special school. She has strabism and visual impairment. Growth was poor in early childhood but since six years of age her weight and length is within normal range. Severe obstipation and sleeping difficulties are recurrent problems. Magnetic resonance imaging (MRI) of the brain at 2.5 and 9 years of age showed slightly reduced supratentorial white matter volume, but normal degree of myelination for her age. Lactate has been increased in blood (maximum 3.4 mmol/L, ref.<2.3) and cerebrospinal fluid (CSF) (2.9 mmol/L, ref.<2.3). Tau protein in CSF is slightly increased (308 ng/L, ref. <250), indicating neuronal damage. In a muscle biopsy, performed at 19 months, ATP production and respiratory chain (RC) enzyme activities were normal (Figure S1a,b).

Subject 2 (II:2), the younger sister, was delivered by cesarean section in gestational week 33, due to IUGR. Her BW was 1655 g, BL 41 cm. She has had a similar disease course to her sister, with developmental delay seen from first year of life, axial hypotonia with spastic arms and legs, ataxia, and strabism. She has no visual impairment and, so far, no epilepsy. At the current age of 10.5 years she has severe intellectual disabilities. Growth is within the lower normal range. Like her sister, she suffers from obstipation and sleeping difficulties. She also has a minimal ASD that does not require surgical correction. MRI of the brain at 2.5 years showed a more severe loss of supratentorial white matter, compared to her sister. Lactate in blood and CSF has been normal. ATP production and RC enzyme activities in a muscle biopsy obtained at 2.5 years were normal (Figure S1a,b).

## 2 RESULTS

### 2.1 Identification of two compound heterozygous *WARS2* variants

Whole exome sequencing (Stranneheim et al., 2014) on genomic DNA samples from both subjects and their parents, followed by Sanger sequencing, identified the two compound heterozygous variants c.833T>G (p.Val278Gly) and the previously reported c.938A>T (p.Lys313Met) (Theisen et al., 2017; Vantroys et al., 2018; Wortmann et al., 2017) in the *WARS2* gene (HGNC:12730) as potential disease‐causing variants (Figure 1a,b). Variants in other genes were dismissed as they failed to show an appropriate inheritance model. Both variants are located within the last exon of isoform 1 (NM_015836.3) of *WARS2*, and allele‐specific amplifications on cDNA derived from both individuals showed that this was the only isoform expressed in fibroblasts. Both mutations are within conserved domains of WARS2 (NP_056651.1) (Figure 1c), with population frequencies of 0.0001483 for c.938A>T (p.Lys313Met) and 0.0002439 for c.833T>G (p.Val278Gly), according to the gnomAD database. Bioinformatic prediction tools, such as PolyPhen‐2 (Adzhubei, Jordan, & Sunyaev, 2013) or PROVEAN (Choi & Chan, 2015), predict pathogenicity for both variants (PolyPhen‐2: possibly damaging with a score of 0.708 for both mutations and PROVEAN: cutoff = −2.5, p.Lys313Met = −2.9, p.Val278Gly = −6.6).

### 2.2 The p.Val278Gly and p.Lys313Met variants modeled in *S. cerevisiae* cause a respiratory chain defect

**Figure 2:**
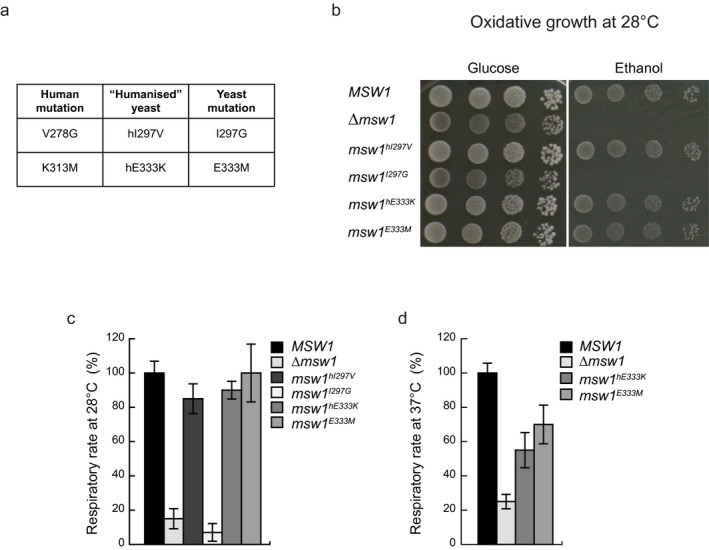
(a) Table indicating the positions and amino acid changes between human WARS2 (NP_056651.1) and yeast Msw1 (NP_010554.1). (b) Growth of KO (∆*msw1*), humanized (*msw1^hI297V^, msw1^hE333K^*), mutant (*msw1^I297G^, msw1^E333M^*), and control (*MSW1*) yeast strains on fermentable (glucose) and nonfermentable (ethanol) carbon sources. (c, d) Respiratory rate of yeast strains indicated under (b) at (c) 28°C and (d) 37°C. Data are represented as mean ± standard deviation (*SD*), *n* = 3 independent experiments.

In order to confirm pathogenicity of the identified variants, we expressed both humanized control (*msw1* ^hI297V^, *msw1* ^hE333K^) and mutant WARS2 (*msw1* ^I297G^, *msw1* ^E333M^) in a yeast strain deleted of the *WARS2* ortholog gene *MSW1* (*∆msw1*) (Figure 2a). Humanized versions were created since the two residues are not conserved, by replacing the yeast aminoacid with the corresponding aminoacid present in the wild type human allele, as previously described (Sommerville et al., 2017). Humanization of *MSW1* did not compromise growth in nonfermentable carbon source and was able to rescue the *∆msw1 *strain (Figure 2b). WARS2‐deficient yeast, expressing the p.Val278Gly (p.I297G in yeast) variant, completely failed to grow under these conditions at 28°C, supporting pathogenicity of this variant, while growth of the p.Lys313Met variant (p.E333M in yeast) was not impaired (Figure 2b). Respiration measurements at 28°C revealed a mitochondrial defect in the *msw1* ^I297G^ but not in the *msw1* ^E333M ^strain (Figure 2c). However, incubation at 37°C did reveal respiratory rate defect in both the *msw1* ^E333M^ and *msw1* ^hE333K^ strains (Figure 2d), suggesting that any changes at this position may affect respiration in yeast in a temperature sensitive manner.

### 2.3 *WARS2* variant causes specific OXPHOS defect in neuroepithelial‐like stem cells

**Figure 3:**
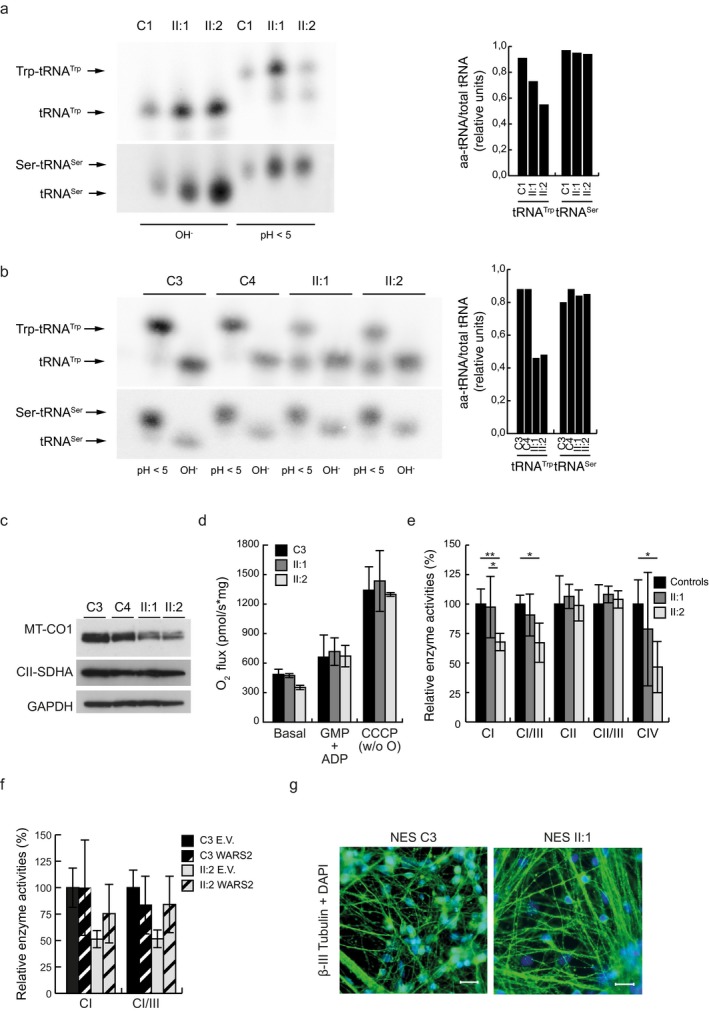
(a) Left: representative acidic‐UREA PAGE followed by Northern blot analysis indicates the aminoacylated (Trp‐tRNA^Trp^, Ser‐tRNA^Ser^), versus uncharged (tRNA^Trp^, tRNA^Ser^) tRNA under acidic (pH < 5) or basic (OH‐) conditions in fibroblasts of subjects (II:1 and II:2) and one nonrelated control (C1). Right: quantification of (a) under acidic (pH < 5) conditions. (B) Left: representative acid‐urea PAGE followed by Northern blot analysis indicates the aminoacylated (Trp‐tRNA^Trp^, Ser‐tRNA^Ser^) versus uncharged (tRNA^Trp^, tRNA^Ser^) tRNA in acidic (pH < 5) or basic (OH^‐^) conditions in NES cells of subjects (II:1 and II:2) and nonrelated controls (C3 and C4). Right: quantification of aminoacylated tRNA^Trp^ of (b) in acidic condition (pH < 5). (c) Representative Western blot analysis of mitochondrial encoded complex IV subunit COI and nuclear encoded complex II subunit A from whole protein extracts of NES cells in nonrelated controls (C3 and C4) and subjects (II:1 and II:2). GAPDH was used as a loading control (*n* = 2 independent experiments). (d) Oxygen consumption rates in subjects (II:1 and II:2) and nonrelated control (C3) using glutamate, malate, and pyruvate (GMP + ADP) as electron donors or by uncoupling the membrane potential with CCCP after succinate injection. Data are normalized to protein content in each sample and represented as mean ± standard deviation (*SD*) (*n* = 3 independent experiments). (e) Relative enzyme activities of respiratory chain enzyme complex I (NADH coenzyme Q reductase), complex I/III (NADH cytochrome c reductase), complex II (succinate dehydrogenase), complex II/III (succinate:cytochrome *c* reductase, SCR), and complex IV (cytochrome *c* oxidase) in isolated mitochondria of subjects (II:1 and II:2) and nonrelated controls (C3 and C4). Data are represented as mean ± standard deviation (*SD*), *p < 0.05, **p < 0.01 *n = *5 independent experiments. (f) Relative enzyme activities of respiratory chain enzyme complex I (NADH‐coenzyme Q reductase) and of CI/III ratio in isolated mitochondria of subject II:2 and C1 control, nucleofected with either pIRES‐eGFP (E.V.) or a pIRES‐eGFP‐WARS2 construct. Data are represented as mean ± standard deviation (*SD*), *n* = 3 independent experiments. (g) Representative immunostaining analysis with DAPI (blue) and beta‐III tubulin (green) of neurones derived from subject (II:1) and control (C3) NES cells. Scale bars 20 µm.

Fibroblasts from subjects II:1 and II:2 were isolated and cultured on glucose or galactose media. In agreement with results obtained in muscle, fibroblasts did not show compromised mitochondrial respiration (Figure S2a), despite showing a strong reduction in fully aminoacylated tRNA^Trp ^(Figure 3a).

Mutations in *ARS2* genes seem to predominantly lead to symptoms originating from the central nervous system, and we therefore re‐programmed patient fibroblasts to iPS cells and further differentiated them to neuroepithelial‐like stem (NES) cells. NES cells exhibit an extensive proliferative lifespan and display a high differentiation potential to various neuronal subtypes and glial cells, allowing for a cell‐type and patient‐specific characterisation (Falk et al., 2012; Koch, Opitz, Steinbeck, Ladewig, & Bruestle, 2009). A substantial proportion of tRNA^Trp^ from both subjects (NES^II:1^, NES^II:2^), when separated under acidic conditions, was not aminoacylated (Figure 3a,b). Western blot analysis of the mitochondrial‐encoded subunit MTCOI revealed reduced levels in both patient‐derived lines, while the nuclear‐encoded complex II subunit SDHA was unaffected (Figure 3c), which is consistent with a defect in mitochondrial gene expression. Furthermore, we observed a mild reduction in basal oxygen consumption (Figure 3d) and decreased activity of respiratory chain complexes I and IV (Figure 3e) in NES^II:2 ^cells. Aminoacylation (Figure S3a) and respiratory chain enzyme complex activities (Figure 3f) could be rescued by expressing a control WARS2 construct in NES^II:2 ^cells (Figure S3b), strongly indicating pathogenicity of the endogenous variants. Despite these defects, NES cells carrying the *WARS2* mutations were able to initiate differentiation into neuronal progenitor cells (Figure 3g).

### 2.4 WARS2 depletion causes lethality and OXPHOS deficiency in *Drosophila melanogaster*

**Figure 4:**
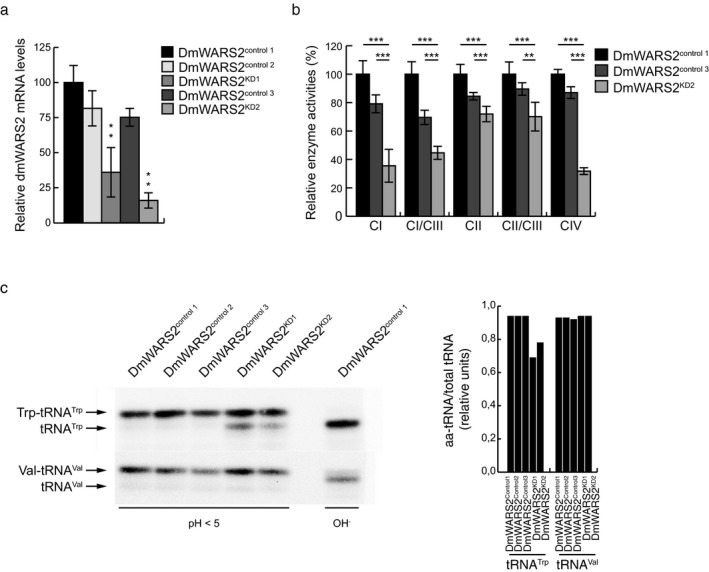
*DmWARS2* knockdown in *Drosophila*. (a) Relative *DmWARS2* mRNA levels in third‐instar larvae of *dmWARS2* KD lines (KD1 and KD2) and controls (control 1, control 2, and control 3). Data are represented as mean ± standard deviation (*SD*) (**p < 0.01, *n = *5 independent experiments). (b) Relative enzyme activities of respiratory chain enzyme complex I (NADH coenzyme Q reductase), complex I/III (NADH cytochrome c reductase), complex II (succinate dehydrogenase), complex II/III (succinate cytochrome *c *reductase), and complex IV (cytochrome *c* oxidase) in isolated mitochondria from *dmWARS2 *KD line (KD2) and controls (control 1 and control 3) larvae. Data are represented as mean ± standard deviation (*SD*) (**p < 0.01, ***p < 0.001, *n = *5 independent experiments). (c) Left: representative acidic‐UREA PAGE followed by Northern blot analysis indicates the aminoacylated (Trp‐tRNA^Trp^, Val‐tRNA^Val^), versus uncharged (tRNA^Trp^, tRNA^Val^) tRNA under acidic (pH < 5) or basic (OH‐) conditions in third‐instar larvae of *dmWARS2* KD lines 1 and 2 (DmWARS2^KD1^ and DmWARS2^KD2^) and control lines 1, 2, and 3 (DmWARS2^control1 ^DmWARS2^control2 ^DmWARS2^control3^). Right: quantification of (a) under acidic (pH < 5) conditions.

The aminoacylation status of mitochondrial tRNAs varies greatly between tRNAs, tissues, and metabolic state and we therefore wanted to see whether a partial defect in tRNA^Trp^ aminoacylation was compatible with life. We therefore silenced *WARS2* gene expression in *Drosophila melanogaster* (Dm), using two independent RNAi lines and the GAL4‐UAS expression system. Loss of DmWARS2 (Figure 4a) caused larval or pupal lethality, with a severe reduction in oxygen consumption (Figure S4) and respiratory chain enzyme activities (Figure 4b). Interestingly, aminoacylation of tRNA^Trp^ was partially affected (Figure 4c), which is in similarity with the aminoacylation defect observed in subjects II:1 and II:2, supporting that a partial defect is sufficient to result in a mitochondrial dysfunction.

## 3 DISCUSSION

Due to the central importance of mitochondria, disruption of respiration would be expected to affect the cellular energy production in all cells equally. However, especially children often present with a progressive neurological disorder, and currently only limited treatment options exist. A growing number of mitochondrial disorders are caused by primary defects in the mitochondrial translation machinery, originating either in mtDNA‐encoded genes, such as tRNAs or rRNAs (Nunnari & Suomalainen, 2012) or nuclear‐encoded subunits of the mitochondrial ribosome or tRNA modification enzymes (Fernández‐Vizarra, Berardinelli, Valente, Tiranti, & Zeviani, 2007; Kopajtich et al., 2014; Lake et al., 2017).

The golden standard in clinical diagnosis of defects in oxidative phosphorylation is measuring respiratory chain function in skeletal muscle tissue specimens. A severe limitation with this approach is that it does not adequately capture defects specifically affecting the central nervous system.

We here report the functional validation of two mutations in the mitochondrial tryptophanyl‐tRNA synthetase gene, leading to a progressive neurodegeneration in early infancy.

A growing number of mutations variants in *WARS2* have been associated with mitochondrial disease (see Table 1 and recently summarized in Vantroys et al. [2018]), with the first described in 2017. Musante et al. described two siblings with severe intellectual disability, ataxia, speech impairment, athetosis, and aggressive behavior due to a frameshift mutation and a p.Trp13Gly mutation in the mitochondrial localization signal that the authors demonstrated to affect mitochondrial localization of the mature WARS2 protein (Musante et al., 2017). Theisen and colleagues reported in the same year a male patient with severe infantile onset of leukocephalopathy, spastic quadriplegia, and epilepsy. The clinical presentation was attributed to the compound heterozygous mutations p.Leu100del and p.Lys313Met. Patient fibroblasts had reduced de novo mitochondrial protein synthesis, reduced steady‐state levels in mitochondrial respiratory chain subunits, and reduced basal and maximal respiration in patient fibroblasts, consistent with deficiencies in mitochondrial translation (Theisen et al., 2017).

**Table 1:** Clinical and molecular findings of all reported WARS2 patients

Mutation	Reference	Clinical findings	Molecular findings
Trp13Gly Ser109Alafs*15	F2‐V:7 in Musante et al. (2017)	Ataxia and athetosis Aggressive behaviour Intellectual disability	Reduced colocalization of mutated (Trp13Gly) WARS2 with mitotracker or HSP60 in HEK293T Cell fractionation shows impaired localization of mutated (Trp13Gly) WARS2 in HEK293T
Trp13Gly Ser109Alafs*15	F2‐V:8 in Musante et al. (2017)	Ataxia and athetosis Aggressive behavior Intellectual disability	Reduced colocalization of mutated (Trp13Gly) WARS2 with mitotracker or HSP60 in HEK293T Cell fractionation shows impaired localization of mutated (Trp13Gly) WARS2 in HEK293T
Leu100del Lys313Met	Theisen et al. (2017)	Microcephaly Leukoencephalopathy Spastic quadriplegia Hypoglycemia (neonatal) Epilepsy Intellectual disability	Reduced mitochondrial translation in fibroblasts Decreased levels of CI, CIII, and CV subunits in fibroblasts Decreased oxygen consumption rates (OCR) in fibroblasts
Lys31_Gln116del Val349Leu	F1, I1 in Wortmann et al. (2017)	Lactic acidosis	Reduced CI and COX staining in liver but not in muscle Normal enzyme activities in fibroblasts Reduced levels of WARS2 and CO2 subunit of CIV in isolated mitochondria from fibroblasts
Pro266Argfs*10 Lys313Met	F2, I2 in Wortmann et al. (2017)	Lactic acidosis Epilepsy Hypoglycemia	Reduced Trp aminoacylation in fibroblasts Decreased mtTrpRS stability in fibroblasts and muscle Normal enzyme activities in fibroblasts
Pro266Argfs*10 Lys313Met	F2, I3 in Wortmann et al. (2017)	Lactic acidosis Epilepsy Hypoglycemia (neonatal)	Reduced Trp aminoacylation in fibroblasts Decreased mtTrpRS stability in fibroblasts and muscle Normal enzyme activities in fibroblasts but reduced CII + III in muscle
His77Gln Glu352Lys	F3, I4 in Wortmann et al. (2017)	Epilepsy Muscular hypotonia Cardiomyopathy Retinitis pigmentosa	Reduced levels of WARS2 and CO2 subunit of CIV in isolated mitochondria from fibroblasts Normal enzyme activities in fibroblasts Reduced activity of all complexes in muscle
Val178Leu (homozygous)	F4, I5 in Wortmann et al. (2017)	Dystonia Suspected epilepsy	
Gly45Val Lys313Met	F5, I6 in Wortmann et al. (2017)	Ataxia Nystagmus	Reduced CI+III activity in muscle
Trp13Gly Ser228Trp	Burke et al. (2018)	Parkinson‐like symptoms Dystonia	Decreased WARS2 expression, but normal expression of mitochondrial complexes in fibroblasts Normal histology and COX/SDH staining and enzyme activities in muscle Increased CoQ10 levels in muscle Reduced mtDNA levels in muscle
Pro266Argfs*10 Lys313Met	Vantroys et al. (2018)	Epilepsy Ptosis Hypoglycemia Hepatotoxicity	Immunostainings of liver Reduced CIII activity in liver homogenate Reduced CI, CIII, and CIV activities (in‐gel stainings), CV subcomplex in liver Decreased WARS2 expression in liver
Val278Gly Lys313Met	This report	Growth retardation Leukoencephalopathy Muscle weakness Hypotonia Ataxia Epilepsy	Normal mitochondrial activity in muscle and fibroblasts Reduced aminoacylation in fibroblasts and NES Mild reduction in CI and CIV activities in NES Rescue of the aminoacylation defect Disease model in yeast and Drosophila melanogaster

In total, the p.Lys313Met mutation has now been reported in seven individuals from five families with various clinical presentations (Table 1) (Theisen et al., 2017; Vantroys et al., 2018; Wortmann et al., 2017). In three cases the p.Lys313Met mutation was paired with the frameshift mutation p.Pro266Argfs*10 (Vantroys et al., 2018; Wortmann et al., 2017), while an additional individual was reported to carry a p.Gly45Val substitution (Wortmann et al., 2017). The effect of the p.Lys313Met mutation on WARS2 function is not clear, as no in vitro studies have been performed. Wortmann and colleagues demonstrate reduced WARS2 levels in liver, resulting in an aminoacylation defect of tRNA^Trp^, in a patient with the p.Lys313Met mutation, which was in compound with a frameshift mutation. By modeling the p.Lys313Met mutation in yeast we demonstrate that this position seems to have some functional importance as both the patient mutation, as well as the humanization of the yeast variant resulted in reduced respiration at 37°C. Moreover, we also observed partial aminoacylation of tRNA^Trp^, both in patient fibroblasts and NES cells, further advocating pathogenicity of this variant. In contrast, the p.Val278Gly substitution reported here has not been previously associated with mitochondrial disease, and its potential effect on WARS2 function is not immediately clear. However, modeling the mutation in yeast resulted in a severe defect on OXPHOS growth.

Unlike patient fibroblasts carrying the p.Lys313Met mutation with the p.Leu100del (Theisen et al., 2017), we did not observe a OXPHOS defect in fibroblasts grown in galactose, nor in a skeletal muscle biopsy. However, reprogramming of primary fibroblasts from either patient to NES cells confirmed the aminoacylation defect observed in fibroblasts, and in addition revealed reduced basal respiration and reduced complex I and IV activities in at least one of the patients’ cell lines analyzed. Aminoacylation levels of tRNA^Trp^ were slightly higher in patient fibroblasts in comparison to patient‐derived NES cells, possibly indicating that neuronal tissues are more sensitive to ARS2 defect. NES cells express a variety of markers of foetal brain development, but can still be cultured and expanded to large volumes without losing their differentiation potential (Falk et al., 2012; Koch et al., 2009), making them a suitable model to study mitochondrial diseases with primarily brain involvement. However, more work will be needed in order to unravel why the brain and NES cells are more sensitive to ARS2‐induced defects of mitochondrial translation compared to other tissues.

The two variants reported here resulted in a partial aminoacylation defect of the mitochondrial‐encoded tryptophan tRNA. By silencing DmWARS2 we were able to demonstrate that such a partial reduction in aminoacylation was sufficient to severely affect fly development. Similarly, compromised ARS2 gene expression in flies has previously been shown to be able to model mitochondrial disease (Bayat et al., 2012; Guitart, Picchioni, Piñeyro, & Ribas de Pouplana, 2013; Meiklejohn et al., 2013).

Although different clinical presentations are often reported for different *ARS2* genes, they are usually quite similar within the same *ARS2* gene (Sissler et al., 2017). For instance, mutations in *DARS2* (Scheper et al., 2007) and *EARS2* (Steenweg et al., 2012) are associated with leukoencephalopathy, not necessarily seen in the clinical presentations of patients with other *ARS2* variants. However, the clinical spectrum of *WARS2* mutations seems to be quite broad, including fatal and nonfatal neonatal lactic acidosis, infantile leukoenchephalopathy, spastic quadriplegia, epilepsy, microcephaly, failure to thrive, developmental delay, intellectual disability, ataxia, athetosis, and muscular hypotonia (summarized in Table 1). Additionally, Vantroys and colleagues reported hepatopathy, possibility triggered by sodium valproate treatment (Vantroys et al., 2018), while the patient reported by Burke and colleagues was affected by Parkinson‐like symptoms from the age of 1 year onwards (Burke et al., 2018). Both patients reported here were born prematurely due to IUGR and had a congenital heart defect. Whether this is attributable to the *WARS2* variants is unclear. Varying responses to different *ARS2* mutations might be explained by different tissue‐specific responses to ARS2 mutations as exemplified by a mouse model for DARS2 (Dogan et al., 2014), or by differences in the compatibility between the *ARS2* variants and their corresponding tRNAs (Meiklejohn et al., 2013; Perli et al., 2016; Wang et al., 2016). Additionally, besides the primary defect of reduced aminoacylation, secondary metabolic defects, such as the accumulation of aminoacyl intermediates, such as tryptophan or tryptamine, an intermediate of tryptophan metabolism and potentially a neurotransmitter, may further affect brain function. These, and other cell‐type and genotype‐specific responses, will therefore affect the clinical presentation and studying neuro‐specific cell models, such as NES cells with different ARS2 variants, might be an important tool to identify at least some of these factors involved in ARS2 diseases.

## 4 MATERIALS AND METHODS

### 4.1 Ethical considerations

Approval for the patient investigations was obtained from the regional Ethics Committee at the Karolinska Institute, Sweden (2014/995‐32). All patients were given standard care and the study was conducted according to the Declaration of Helsinki. Written informed consent for this study was obtained from the guardians of all included patients and controls.

### 4.2 Cell culture

Human primary skin fibroblasts (passage 4–15 from patients II:1 and II:2 as well as control C1 and C2) were cultured at 37°C/5% CO_2_ atmosphere in standard high‐glucose DMEM‐Glutamax media (Thermo Fisher Scientific), supplemented with 10% FBS (Thermo Fisher Scientific), or in glucose‐free DMEM‐Glutamax media, supplemented with 5 mM galactose and 10% dialysed FBS. Experiments were performed with cells cultured to 80%–90% confluency.

Fibroblasts from subjects II.1, II.2, and controls (C3, a healthy male individual and C4, a healthy female individual) were reprogrammed to iPS cells as previously described (Kele et al., 2016; Uhlin et al., 2017), using integration‐free Sendai virus vector mediated reprogramming (CytoTune™‐iPS 2.0 reprogramming kit, Thermo Fisher Scientific), before undergoing neural lineage induction (Chambers et al., 2009) and NES cell isolation (Falk et al., 2012; Koch et al., 2009).

NES cells (passage 15–50) were cultured as previously described (Falk et al., 2012), on 100 μg/ml poly‐ornithine/2 μg/ml laminin‐coated culture dishes (Sigma‐Aldrich) at 37°C/5% CO_2_ atmosphere in DMEM/F‐12 Glutamax (Thermo Fisher Scientific) media, containing 17 mM glucose, 100 units/ml penicillin‐streptomycin (Thermo Fisher Scientific), N‐2 (1:100) (Thermo Fisher Scientific), B27 (1:1,000) (Thermo Fisher Scientific), 10 ng/μl EGF (Peprotech), and 10 ng/μl bFGF (Thermo Fisher Scientific). Cells were trypsinized using TrypLE Express 1x (Thermo Fisher Scientific). Neuronal differentiation was induced by excluding the growth factors bFGF and EGF from the media and increasing the concentration of B27 to 1:100. In both conditions, two‐thirds of the culture media was exchanged daily.

NES cells were nucleofected with 2 μg plasmid according to Lonza protocol (AmaxaTM 4D‐NucleofectorTM Basic Protocol for Primary mammalian neurons). For 4D‐NucleofectorTM X Unit‐transfection in suspension. 4 × 10^6 ^cells were used per nucleofection.

### 4.3 Immunostaining and confocal microscopy

Neurones were cultured on laminin‐coated 1,5 mm cover‐slips and fixed for 20 min using 3% paraformaldehyde (VWR) at room temperature followed by permeabilization and blocking, using 0.1% saponin and 1% BSA (Sigma‐Aldrich) in PBS for 1 hr. Immunostaining with beta‐III Tubulin (#T2200, Sigma‐Aldrich) was performed overnight, while samples were incubated with the secondary antibody Alexa Fluor® 488 (Thermo Fisher Scientific) for 1 hr at a 1:1,000 dilution. Slides were mounted with ProLong® Diamond Antifade Mountant (Thermo Fisher Scientific) and 0.01 μg/ml DAPI (Sigma‐Aldrich). A Carl Zeiss fluorescent microscope was used for image acquisition.

### 4.4 Yeast

The W303‐1B genotype is Matα ade2‐1 leu2‐3, 112 ura3‐1 trp1‐1 his3‐11, 15 can1‐100. All experiments except transformation were performed in synthetic complete (SC) media (0.69% YNB without amino acids powder, ForMedium) supplemented with 1gr/L dropout mix (Baruffini, Ferrero, & Foury, 2010) without amino acids or bases necessary to keep plasmids (i.e. uracil for pFL38 and tryptophan for pFL39). Media were supplemented with various carbon sources at 2% (w/v) (Carlo Erba Reagents), in liquid phase or after solidification with 20 g/L agar (ForMedium). Transformation was performed according to Gietz and Schiestl (Gietz & Schiestl, 2007) after growth in YPAD medium (1% Yeast extract, 2% Peptone, 40 mg/L adenine base, and 2% glucose).

*MSW1*, the yeast ortholog of *WARS2*, was cloned under its natural promoter by PCR‐amplification and inserted into the pFL38 vector (Bonneaud et al., 1991). The pFL38‐*MSW1* plasmid was introduced into the W303‐1B strain through the Li‐Ac method (Gietz & Schiestl, 2007) and disruption of the genomic *MSW1* gene was performed in this strain, since the deletion leads to mitochondrial DNA instability (Myers, Pape, & Tzagoloff, 1985). The disruption was performed through one‐step gene disruption by PCR‐amplification of KanMX4 cassette (Wach, Brachat, Pöhlmann, & Philippsen, 1994) from the BY4741 deleted strain, using appropriate primers and transformation of the former strain; thus obtaining W303‐1B*Δmsw1*/pFL38‐*MSW1*. MSW1 fragment was subcloned from pFL38 to pFL39 (Bonneaud et al., 1991) and mutagenized by PCR overlap technique (Ho, Hunt, Horton, Pullen, & Pease, 1989) with appropriate primers to obtain the humanized and mutant alleles, and subsequently cloned into the pFL39 vector.

W303‐1B*Δmsw1*/pFL38‐*MSW1* was transformed with the pFL39 vector carrying the wild type (*MSW1*) or the humanized (*msw1^hI297V^, msw1^hE333K^*) or the mutant (*msw1^I297G^, msw1^E333M^*) alleles or with the empty vector as control, and then pFL38‐*MSW1* was lost through plasmid‐shuffling.

For oxidative growth analyses the strains were serially diluted and spotted on SC agar plates (without tryptophan) supplemented with 2% glucose or 2% ethanol. Plates were incubated at 28°C.

Oxygen consumption rate was measured at 30°C from yeast cell suspensions cultured for 18 hr at 28˚C or for 16 hr at 37°C in SC medium supplemented with 0.6% glucose until exhaustion using a Clark‐type oxygen electrode (Oxygraph System Hansatech Instruments England) with 1 ml of air‐saturated respiration buffer (0.1 M phthalate–KOH, pH 5.0), 0.5% glucose.

### 4.5 Drosophila melanogaster maintenance

To achieve ubiquitous *DmWARS2* knockdown, two independent *Dm *lines w;UAS‐*DmWARS2*‐RNAi were obtained from Vienna Drosophila Resource Centre (#28027, #106121) and backcrossed for six generations to a white Dahomey background (W^Dah^). *DmWARS2* silencing was induced by crossing w;UAS‐*DmWARS2*‐RNAi flies to the *Dm *driver line *daughterless* GAL4 (w;;daGAL4). Flies were grown on a standard yeast‐sugar‐agar medium and maintained at 25^°^C and 60% humidity on a 12h:12h light:dark cycle.

### 4.6 RNA extraction and qPCR

Total RNA extraction was performed using Trizol (Thermo Fisher Scientific), following the manufacturer's instructions, followed by DNase‐treatment, using TURBO DNA*‐free* kit (Thermo Fisher Scientific). A total of 250 ng of RNA were reverse‐transcribed using a High‐capacity cDNA Reverse Transcription Kit (Applied Biosystems). QPCR was performed on a Quant Studio 6 Flex instrument using Platinum^TM^ SYBR^TM ^Green qPCR SuperMix‐UDG (Invitrogen, Life Technologies) and gene‐specific primers. Gene expression was normalized to the house‐keeping gene beta‐actin. For *Drosophila *samples*, *RNA from third‐instar larvae was isolated using the ToTALLY RNA^TM^ Kit (Ambion, Life Technologies). Gene expression was normalized to ribosomal protein L32.

For human samples, forward and reverse primers were as follows: 18S: 5′‐GGG TCA TAA GCT TGC GTT GAT‐3′ and 5′‐AGT CAA GTT CGA CCG TCT TCT‐3′; WARS2: 5′‐GAC TGC TGT TCT TCT‐3′; and 5′‐TGA CCA TGC AGG AAA GGA TC‐3′.

For fly samples, forward and reverse primers were as follows: L32: 5′‐CGG ATC GAT ATG CTA AGC TGT‐3′ and 5′‐CGA CGC ACT CTG TTG TCG‐3′; WARS2: 5′‐AGC AGC CGA CAT TAT GCT TT‐3′; and 5′‐GCC ATA GCG ACC GTT ATA AAT TC‐3′.

### 4.7 Analysis of aminoacyl‐tRNAs by gel electrophoresis

Aminoacylation status of mitochondrial tRNAs was analyzed by acidic PAGE, followed by Northern blot analysis, as described (Freyer et al., 2012). Northern blots were analyzed on a Typhoon FLA 7,000 Phosphorimager (GE Healthcare).

For human samples, primers were as follows: tRNA^Trp^: 5′‐GAT CTG GAG TCA GAC GCG‐3′; tRNA^Ser^:5′‐CAA AAA AGG AAG GAA TCG AAC CCC CC‐3′. For fly samples, the oligonucleotide against tRNA^Trp ^was: 5′‐CTT TAT TTA TAG CTT TGA AGG TTA TTA G‐3′ and for tRNA^Val^ was: 5′‐TTT GCA CAA AAA TCT TTT CAA TG‐3′.

### 4.8 Biochemical evaluation of respiratory chain enzyme activities

Biochemical studies were conducted in isolated mitochondria as previously described (Wibom, Hagenfeldt, & Dobeln, 2002). For mitochondrial isolation, fibroblasts and NES cells were homogenized and washed in 320 mM sucrose, 1 mM EGTA, and 20 mM Tris‐HCl pH7.2 with 0,1% BSA. Dm larvae were washed and homogenized in 250 mM sucrose, 2 mM EGTA, and 5 mM Tris pH 7.4 with 5% BSA. Equal amounts of mitochondrial protein concentrations were resuspended in 250 mM sucrose, 15 mM K_2_HPO_4_, 2 mM MgAc_2, _0.5 mM EDTA, and 0.5 g/L BSA, pH 7.2 before determination of respiratory chain enzyme activities. Mitochondrial oxygen consumption measurements of NES cells and Dm were performed on permeabilized cells and third‐instar larvae, using glutamate, malate, pyruvate (GMP) and ADP for NES cells, or glutamate and malate (GM) and ADP for larvae (all from Sigma‐Aldrich) for complex I‐driven oxygen consumption in an Oroboros oxygraph (Oroboros Instruments GmbH). Maximal respiration was measured by titration of mitochondrial uncoupler CCCP after succinate injection.

### 4.9 Western blot analysis

Western blot analyses were performed on whole NES cell protein extracts. In summary, cells were harvested and lysed in RIPA buffer containing 0.1% sodium dodecyl sulfate. Protein extracts were then separated on a 12% NUPAGE acrylamide gel (Invitrogen), transferred to a PVDF membrane (Millipore) and then probed with following antibodies: complex IV subunit COI (Invitrogen 459,600, 1:1,000), complex II subunit A (Abcam ab14715, 1:2,000), and GAPDH (V‐18) (Santa Cruz SC‐20357, 1:1,000). Proteins were ultimately visualized with Western ECL substrate (Bio‐Rad).

### 4.10 Statistical analysis

Parameters in this study were considered normally distributed. Experiments were performed at least three times (if not otherwise stated) and data are presented as mean ± standard deviation (*SD*); an unpaired, two‐tailed *t* test was used to evaluate the differences. Values with *p* < 0.05 were regarded as statistically significant.

## CONFLICT OF INTEREST

The authors declare no conflict of interest.
