# Supplementary material for: Mutations in the mitochondrial tryptophanyl‐tRNA synthetase cause growth retardation and progressive leukoencephalopathy
Source: Mol Genet Genomic Med. 2019 Mar 28;7(6):e654. doi: 10.1002/mgg3.654 (PMC6565557; doi:10.1002/mgg3.654)
Supplement: Supplementary file 1 [file MGG3-7-e654-s001.pdf]

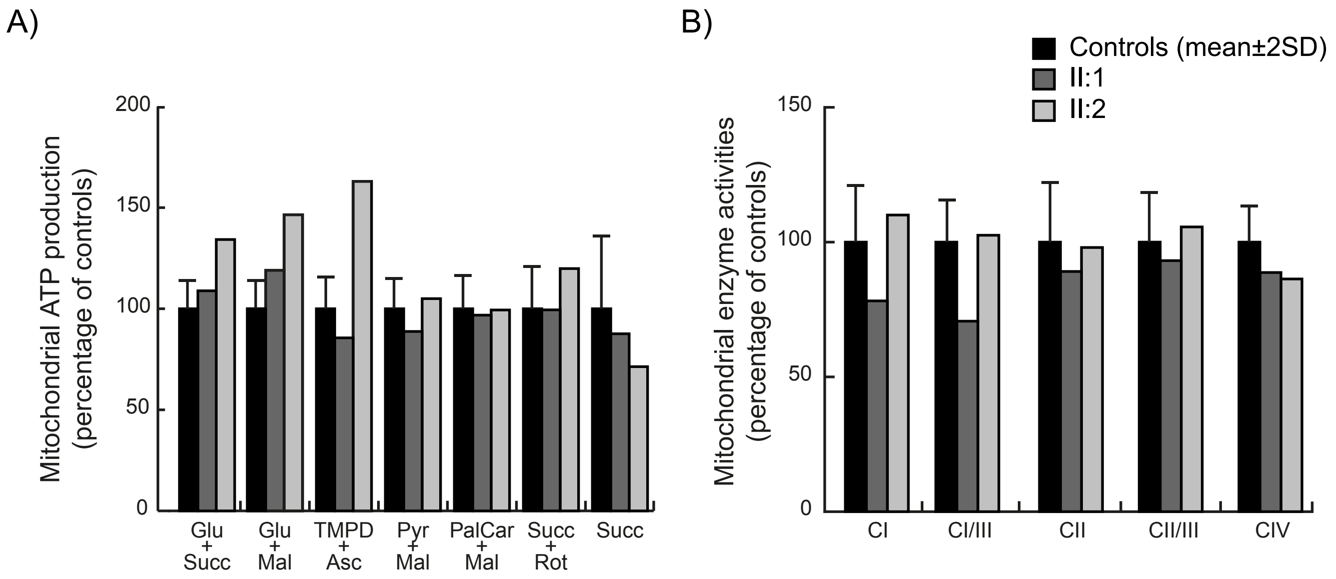

Figure S1. Biochemical analysis of patient muscle biopsies. (A) Mitochondrial ATP production rate in skeletal muscle biopsies from control (n=11; age 0-5 years; black) and subjects II:1 (dark grey) and II:2 (light grey) were determined by a firefly luciferase-based method at 25°C, using glucose and succinate (Glu+Succ), glucose and malate (Glu+Mal), Tetramethyl-p-Phenylenediamine and ascorbate (TMPD + Asc), pyruvate and malate (Pyr + Mal), Palmitoyl carnitine and malate (PalCar + Mal), succinate and rotenone (Succ + Rot) or succinate (Succ) as electron donors. Results are presented as the ATP synthesis rate (units) per unit of citrate synthase (CS) activity (control n=11; age 0–5 years). (B) Respiratory chain enzyme activities of complex I (NADH:coenzyme Q reductase), complexes I and III (NADH:cytochrome c reductase), complex II (succinate dehydrogenase), complexes II and III (succinate:-cytochrome c reductase, SCR), complex IV (COX) and CS were determined. Results are presented as percentage of mean control (n=9; age 0–5 years) values. The range of control values is depicted as ± 2 standard deviations (2SD).

Maffezzini et al. Figure S1
