# Supplementary material for: Mutations in the mitochondrial tryptophanyl‐tRNA synthetase cause growth retardation and progressive leukoencephalopathy
Source: Mol Genet Genomic Med. 2019 Mar 28;7(6):e654. doi: 10.1002/mgg3.654 (PMC6565557; doi:10.1002/mgg3.654)
Supplement: Supplementary file 2 [file MGG3-7-e654-s002.pdf]

A)

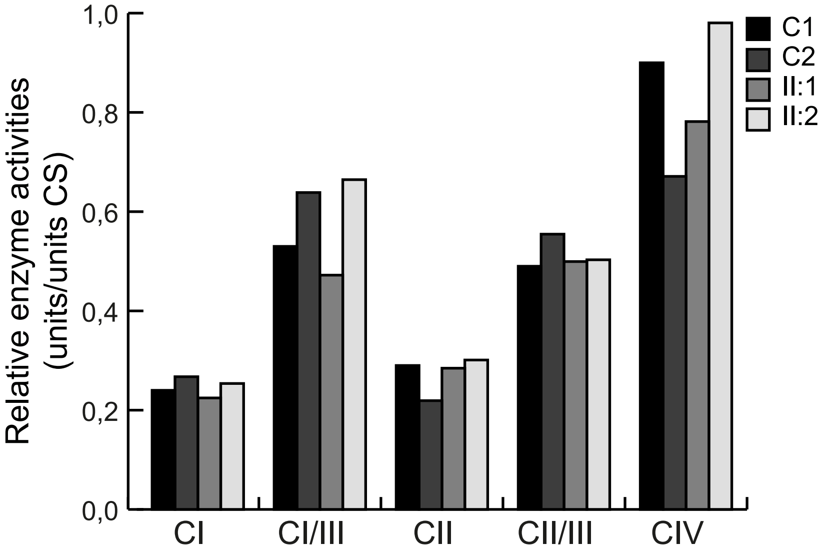

Figure S2. Patient fibroblast fail to show mitochondrial enzymes defects when grown in galactose. (A) Relative enzyme activities of respiratory chain enzyme complex I (NADH coenzyme Q reductase), complex I/III (NADH cytochrome c reductase), complex II (succinate dehydrogenase), complexes II /III (succinate:cytochrome c reductase, SCR) and complex IV (cytochrome c oxidase) in isolated mitochondria of fibroblasts from subjects (II:1 and II:2) and non-related controls (C1 and C2) grown in galactose. Data are represented as mean ± standard deviation (SD), n=1.

Maffezzini et al. Figure S2
