# Supplementary material for: Mutations in the mitochondrial tryptophanyl‐tRNA synthetase cause growth retardation and progressive leukoencephalopathy
Source: Mol Genet Genomic Med. 2019 Mar 28;7(6):e654. doi: 10.1002/mgg3.654 (PMC6565557; doi:10.1002/mgg3.654)
Supplement: Supplementary file 3 [file MGG3-7-e654-s003.pdf]

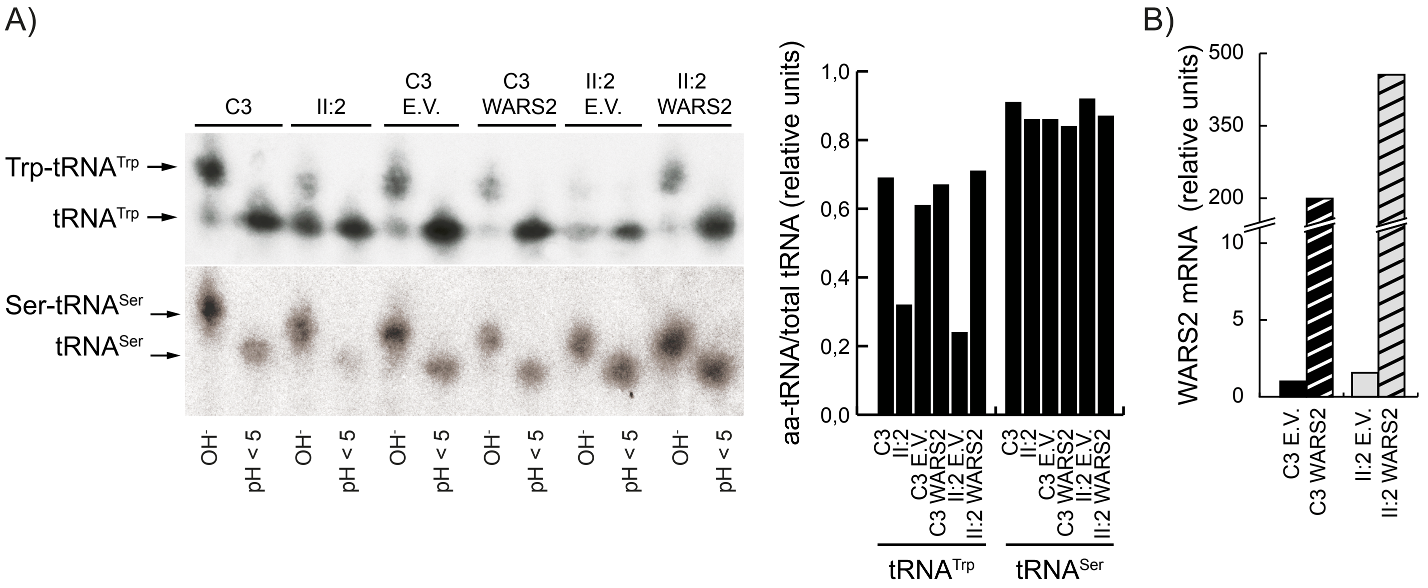

Figure S3. Rescue of the aminoacylation defect in NES cells from subject II:2. (A) Left: Representative acidic-urea PAGE on control (C3), and subject II:2 NES cells transfected with empty pIRES-eGFP (E.V.) or overexpressing WARS2 (WARS2), n=3 independent experiments.

Aminoacylated tRNAs (Trp-$tRNA^{Trp}$, Ser-$tRNA^{Ser}$), migrate slower, when isolated under acidic (pH < 5) conditions. Uncharged tRNAs ($tRNA^{Trp}$, $tRNA^{Ser}$) migrate as seen under basic (OH^-). Right: Quantification of (A) under acidic (pH < 5) conditions. (B) qRT-PCR analysis of WARS2 expression in control (C3) and subject II:2 NES cell lines transfected with either empty pIRES-eGFP (E.V.) or overexpressing WARS2 (WARS2).

Maffezzini et al. Figure S3
