# Supplementary material for: Mutations in the mitochondrial tryptophanyl‐tRNA synthetase cause growth retardation and progressive leukoencephalopathy
Source: Mol Genet Genomic Med. 2019 Mar 28;7(6):e654. doi: 10.1002/mgg3.654 (PMC6565557; doi:10.1002/mgg3.654)
Supplement: Supplementary file 4 [file MGG3-7-e654-s004.pdf]

A)

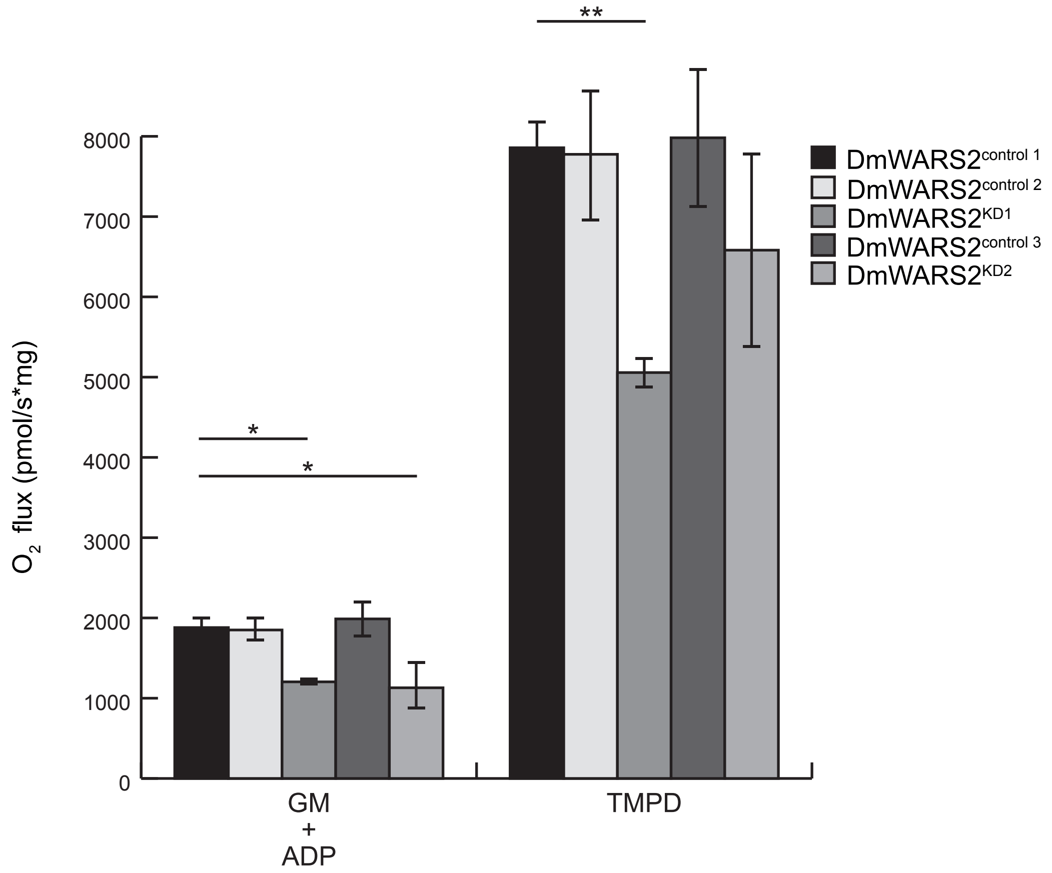

Figure S4. Knock down *WARS2* in *Drosophila melanogaster* leads to decreased oxygen consumption. (A) Oxygen consumption rates in DmWARS2 knock down lines using glutamate and malate (GM + ADP) as electron donors and tetramethyl-p-phenylenediamine (TMPD), an artificial CIV substrate. Data are normalised to the protein content in each sample and are represented as mean ± standard deviation (SD). Differences were analysed by a two-tailed t-test. *P < 0.05, **P < 0.01, n = 6 independent experiments.

Maffezzini et al. Figure S4
